# Supplementary material for: Active thrombin produced by the intestinal epithelium controls mucosal biofilms
Source: Nat Commun. 2019 Jul 19;10:3224. doi: 10.1038/s41467-019-11140-w (PMC6642099; doi:10.1038/s41467-019-11140-w)
Supplement: Supplementary file 1 — Supplementary Information [file 41467_2019_11140_MOESM1_ESM.pdf]

Supplementary information Vergnolle et al.

Active thrombin produced by the intestinal epithelium controls mucosal biofilms

Jean-Paul MOTTA[1#], Alexandre DENADAI-SOUZA[1#], David SAGNAT[1], Laura GUIRAUD[1], Anissa EDIR[1], Chrystelle BONNART[1], Mireille SEBBAG[1], Perrine ROUSSET[1], Ariane LAPEYRE[1], Carine SEGUY[1], Noa MATHURINE-THOMAS[1], Heather J. GALIPEAU[2], Delphine BONNET[3], Laurent ALRIC[3], Andre G. BURET[4], John L. WALLACE[5], Antoine DUFOUR[5], Elena F. VERDU[2], Morley D. HOLLENBERG[5], Eric OSWALD[1], Matteo SERINO[1], Celine DERAISON[1], Nathalie VERGNOLLE[1,5,*]

Author Affiliation:

1. IRSD, Université de Toulouse, INSERM, INRA, ENVT, UPS, U1220, CHU Purpan, CS60039, 31024 Toulouse, France

2. Farncombe Family Digestive Health Research Institute, McMaster University, Health Science Center, Rm 3N4, 1280 Main Street West, Hamilton, Ontario, L8S 4K1 Canada

3. Department of Internal Medicine and Digestive Diseases, 1, avenue Jean Poulhes-TSA 50032, 31059 Toulouse, France

4. Department of Biological Sciences, University of Calgary, 2500 University Drive NW, Calgary, Alberta, T2N 4N1, Canada

5. Departments of Physiology & Pharmacology, and Medicine, University of Calgary Cumming School of Medicine, 3330 Hospital Drive NW, Calgary, Alberta, T2N 4N1, Canada

# These authors contributed equally

***Corresponding Author:** Dr. Nathalie Vergnolle, Inserm UMR-1220, Institut de Recherche en Santé Digestive, CS60039 CHU Purpan, 31024 Toulouse, Cedex-3, France. E-mail address: nathalie.vergnolle@inserm.fr

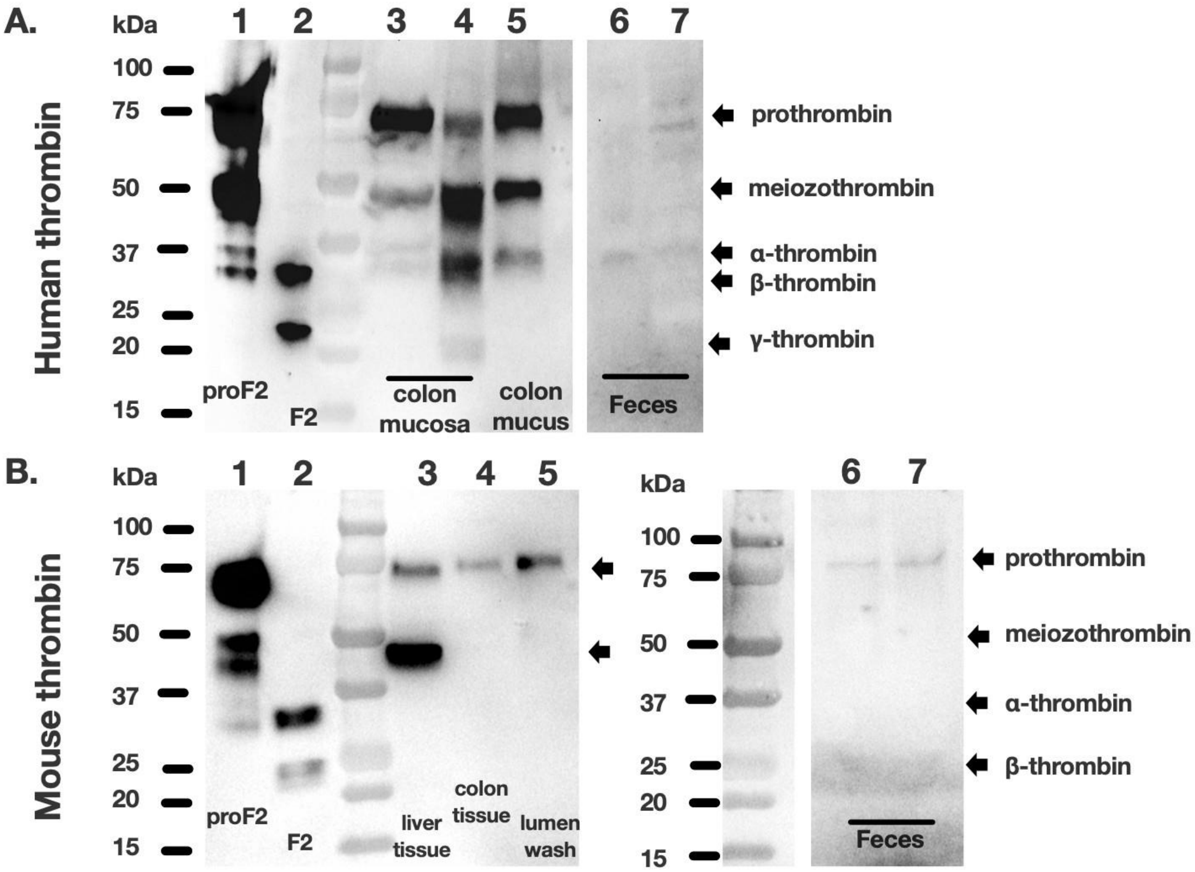

Supplementary Figure 1. Thrombin is present in intestinal lumen of healthy human and mouse. Western blots revealed the presence of prothrombin (75-kDa, black arrows) and its active isoforms (50-kDa, 30-kDa, black arrows) in **A.** human and **B.** mouse intestinal lumen. **A.** Lane 1 is prothrombin, lane 2 is active thrombin, lane 3 is colon mucosa, lane 4 is 24-hours cultured colon mucosa, lane 5 is colon mucus, lane 6 is feces from donor #1, lane 7 is feces from donor #2. **B.** Lane 1 is prothrombin, lane 2 is active thrombin, lane 3 is murine liver tissue, lane 4 is whole colon tissue, lane 5 is whole colon luminal wash, lane 6 is feces from mouse #1, lane 7 is feces from mouse #2. Gaps between ladder and lanes represent irrelevant lanes from the same get cut out. Representative western blots are from 3 independent experiments.

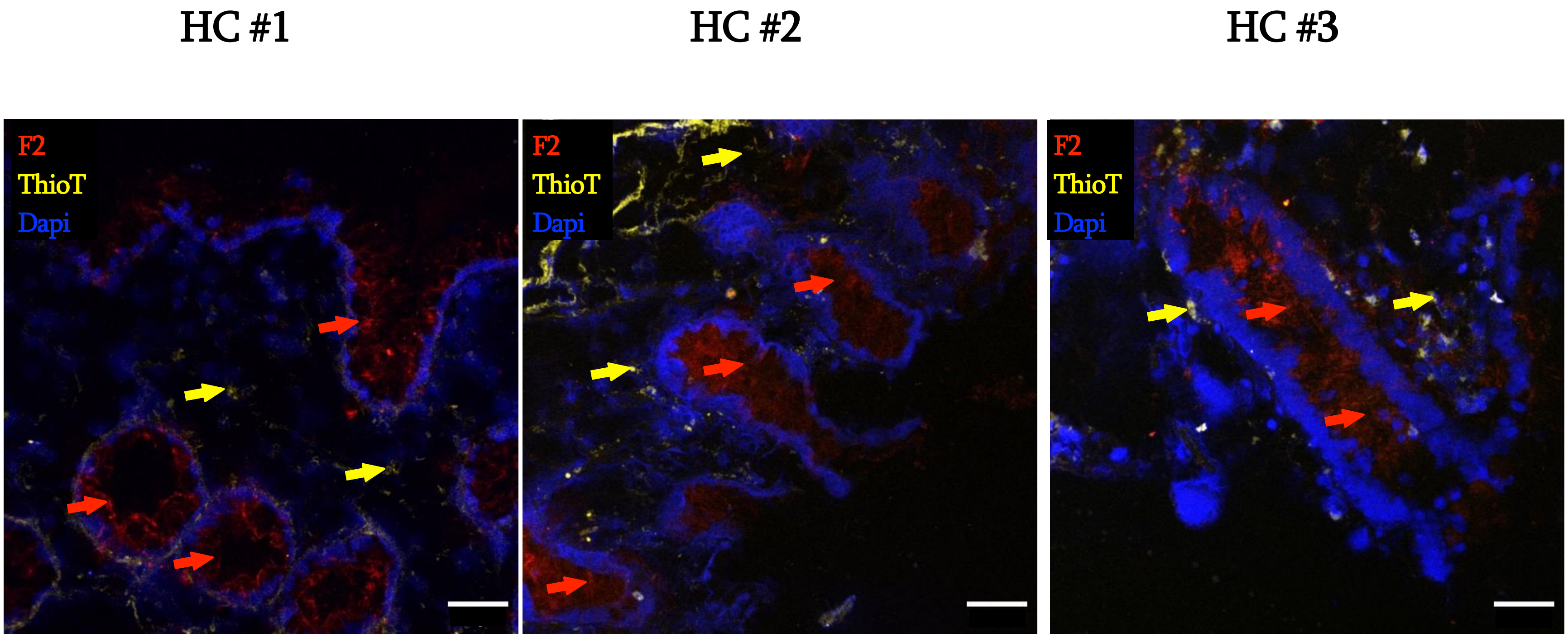

Supplementary Figure 2. Epithelial thrombin does not form or is not associated with amyloid fibril protein aggregates in healthy colon mucosa. Immunostaining of human thrombin (F2, in red) does not colocalize with amyloid-like protein aggregates (Thioflavin T staining, in yellow) in human colon biopsies. Host cell nuclei were stained with 4′,6-diamidino-2-phenylindole or DAPI. Representative images were generated from three different human donors, with at least 3 fields analyzed per individual. Scale bar corresponds to 50 µm.

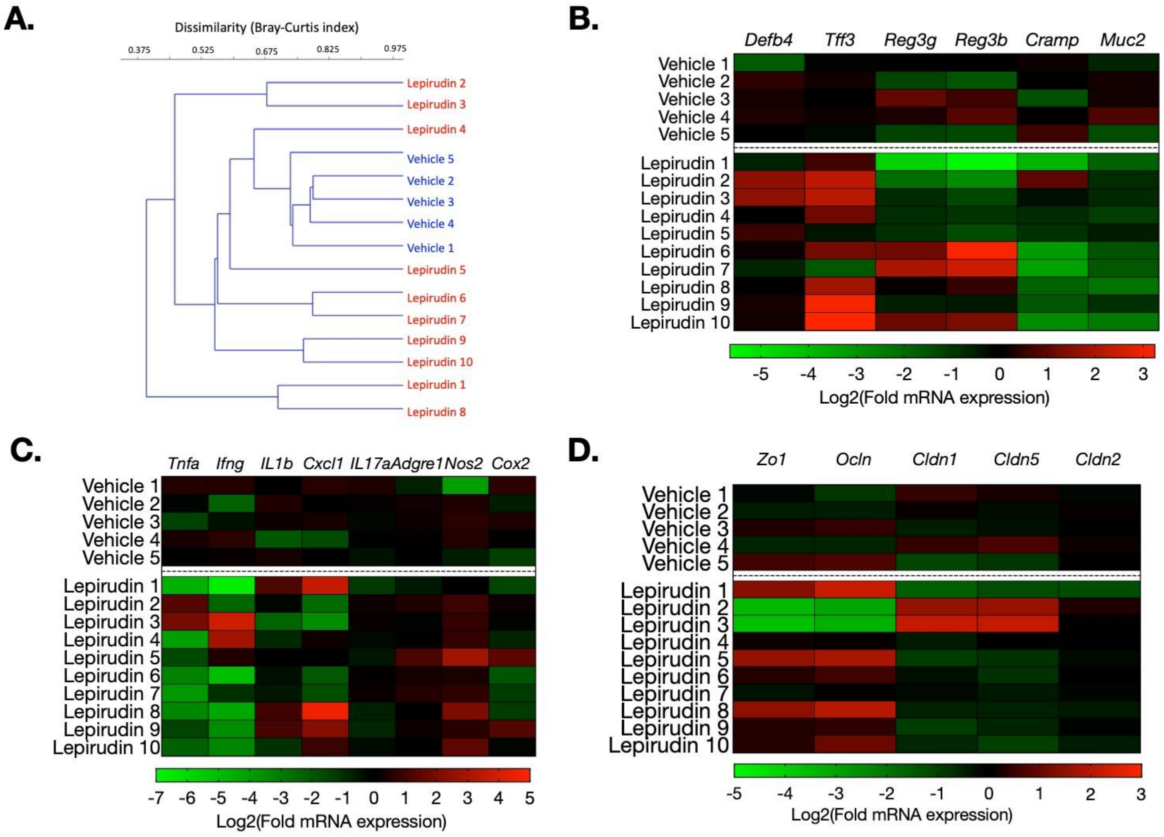

Supplementary Figure 3. Host transcriptome analysis after 10 days administration of vehicle or lepirudin (thrombin inhibitor) in the colon of mice. Transcriptome analysis was performed by qPCR in colon tissue from vehicle- and lepirudin-treated mice. **A.** Hierarchical clustering dendrogram from the Bray-Curtis dissimilarity analysis confirmed a clear separation between lepirudin- and vehicle-treated animals. **B. to D.** Log2 fold changes in mRNA transcription were represented as heat maps (lowest expression in green, highest in red, baseline in black). Transcriptome of host-microbiota related genes (*Defb4, Tff3, Reg3g Reg4b, Camp, Muc2*) is significantly different between lepirudin- and vehicle-treated groups (Permanova with Bonferroni correction P= 0.0241), **C.** but not when considering the transcriptome of inflammatory marker (Co*x2, Nos2, TNFa, IFNg, IL17A, Adgre1, Cxcl1, IL1b,* Permanova with Bonferroni correction P= 0.0949), **D.** nor transcriptome related to tight-junction (*Zo1, Ocln, Cldn1, Cldn2, Cldn5*, Permanova with Bonferroni correction P= 0.553).

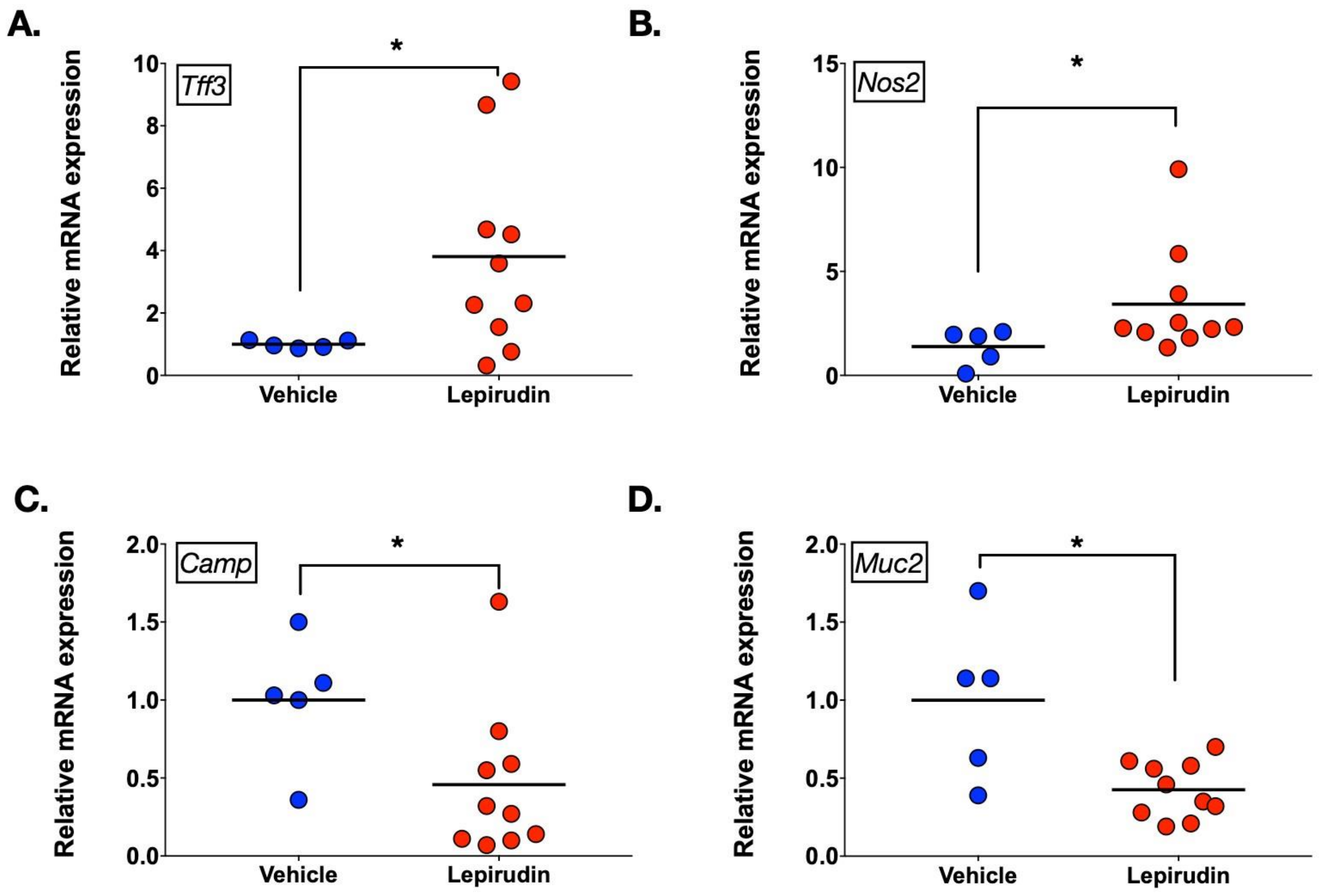

Supplementary Figure 4. Lepirudin treatment caused an altered transcription of genes involved in host-microbiota functions in mice. qPCR gene analysis revealed increased transcription of **A**. *Tff3* and **B.** *Nos2* mRNA, as well as reduced transcription of **C**. *Camp* and **D.** *Muc2* genes in colon of lepirudin- compared to vehicle-treated mice. Data are represented as scatter plot with the mean bar. Unpaired Mann-Whitney test * $P < 0.05$. qPCR analysis has been reproduced twice independently.

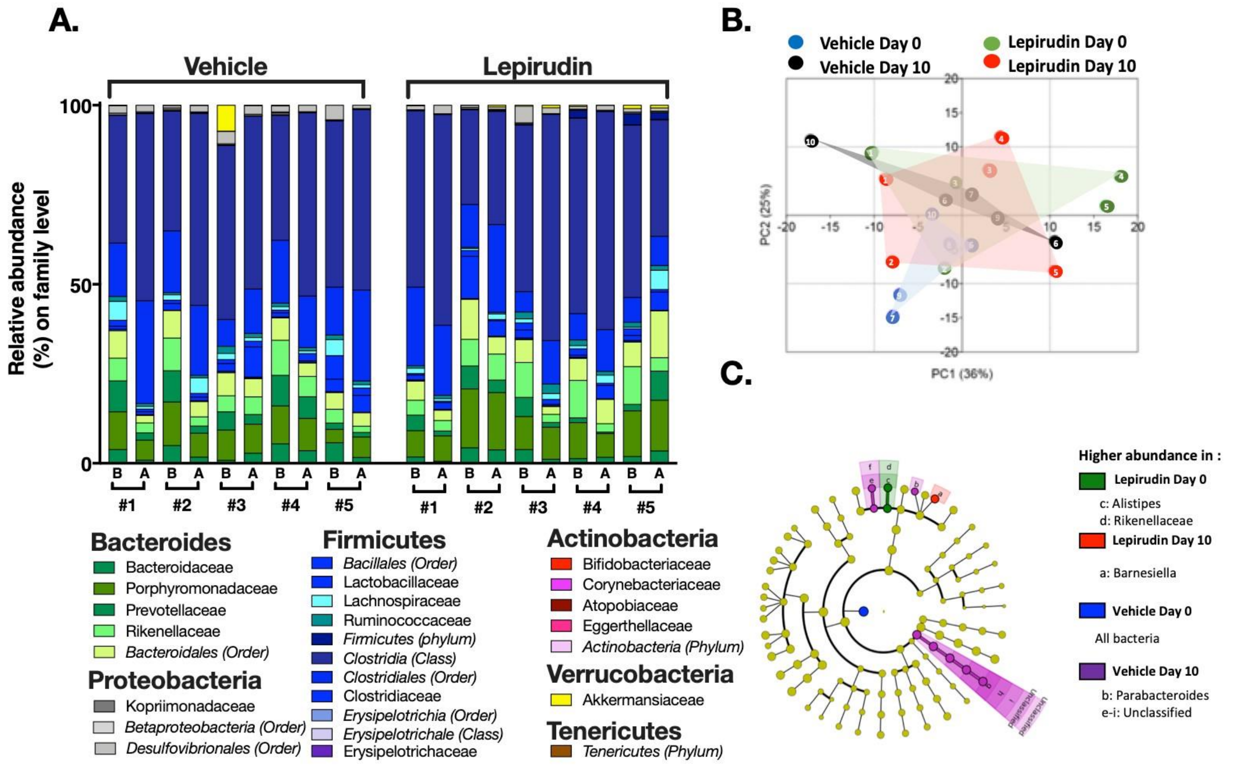

Supplementary Figure 5. Basal thrombin activity inhibition causes minimal alteration on microbiota taxonomy. C57Bl6 mice were treated daily with either vehicle or lepirudin via intracolonic route, and euthanized after 10 days (n=5 per group). Feces were collected at day 0 and at day 10 after lepirudin or vehicle treatment. **A.** 16S Illumina sequencing profiles of bacterial families were determined fecal bacteria from vehicle- and lepirudin treated animals at day 0 (before, B) and day 10 (after, A). **B.** Principal component analysis with filled convex hulls did not reveal a significant shift in overall diversity of fecal microbiota composition between the 4 experimental groups. **C.** Taxonomic cladogram using LEfSe analysis (Linear discriminant analysis effect size) revealed an increased abundance of undetermined Parabacteroides in vehicle-treated group at day 10, and *Alistipes* (Rikenellaceae family) and *Barnesiella* taxons in Lepirudin-treated groups, at day 0 and 10 respectively.

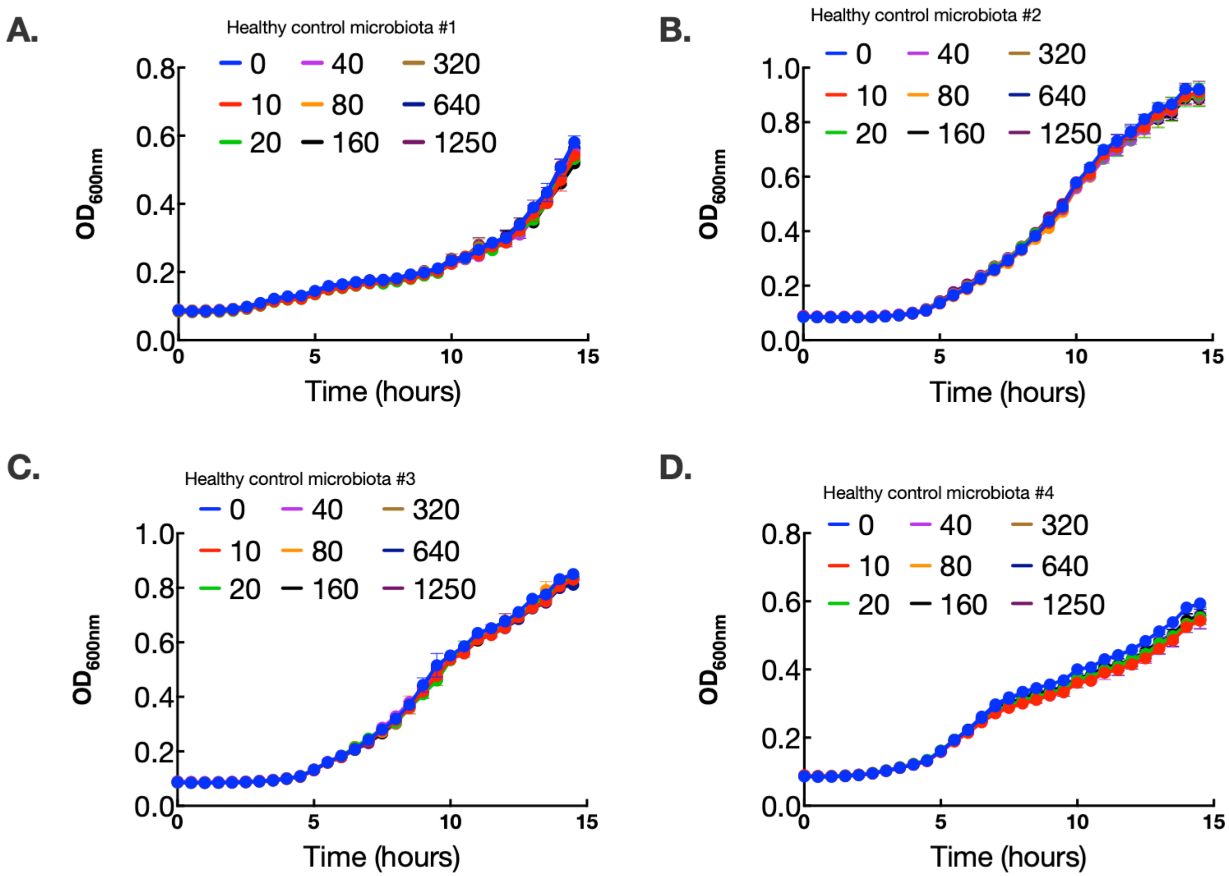

Supplementary Figure 6. Human thrombin is not bactericide nor bacteriostatic on human multispecies microbiota in liquid culture. Mucosa-associated microbiota from 4 human colon biopsies: healthy control microbiota #1, panel **A.**; healthy control microbiota #2, panel **B**; healthy control microbiota #3, panel **C**; healthy control microbiota #4, panel **D**; were diluted in 96-well microplate to optical density of 0.1 ($OD_{600\ nm}$) in M63 media supplemented with glucose (2%), L-cysteine (0.5 %) and containing various concentration of human active thrombin (0 to 1250 $mU.ml^{-1}$, equivalent to 0 to 12.5 nM). The growth curves of each inoculum were generated from OD_{600} reading (20 minutes step) for 15 hours at 37°C, with the plate sealed with a plastic film. N=2 independent experiments.

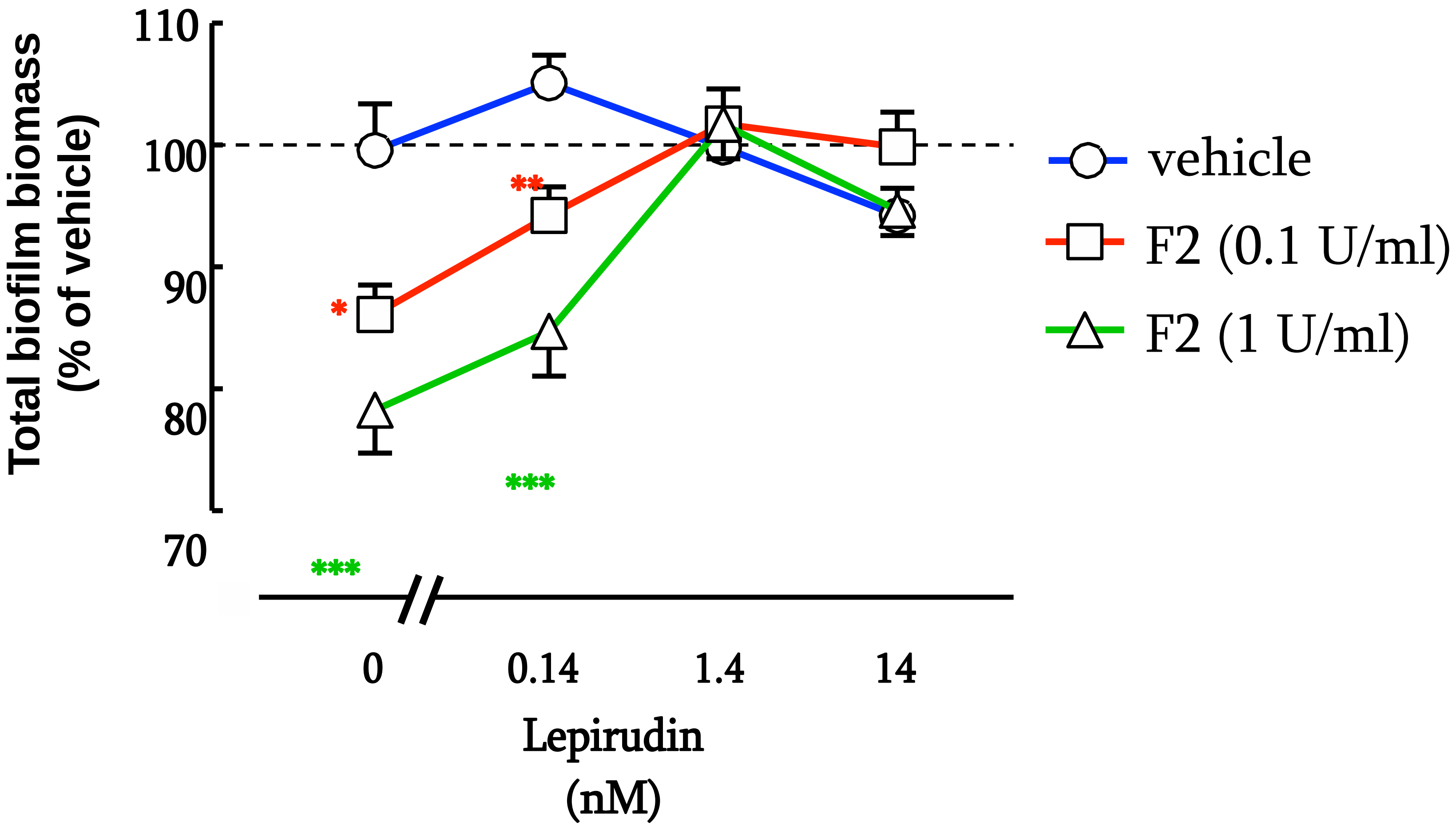

Supplementary Figure 7. Reduction of human multispecies microbiota biofilm biomass by thrombin is dependent on its proteolytic activity. Multispecies anaerobic biofilms were generated from one human healthy donor. Mature biofilms were then exposed to various concentrations of human active thrombin (F2) with or without addition of lepirudin (thrombin irreversible inhibitor). Thrombin, without lepirudin, dose- dependently reduced total biofilm biomass (crystal violet assay). Thrombin-induced effect on biomass was totally abolished when biofilms were incubated with thrombin and lepirudin (1.4 nM and 14 nM). Two-way ANOVA analysis with Fisher's LSD test versus control group no-F2 *P < 0.05, **P < 0.01, ***P < 0.001. Each concentration with 4-16 individual biofilms, N=2

independent experiments. Data are presented as mean ± SEM.

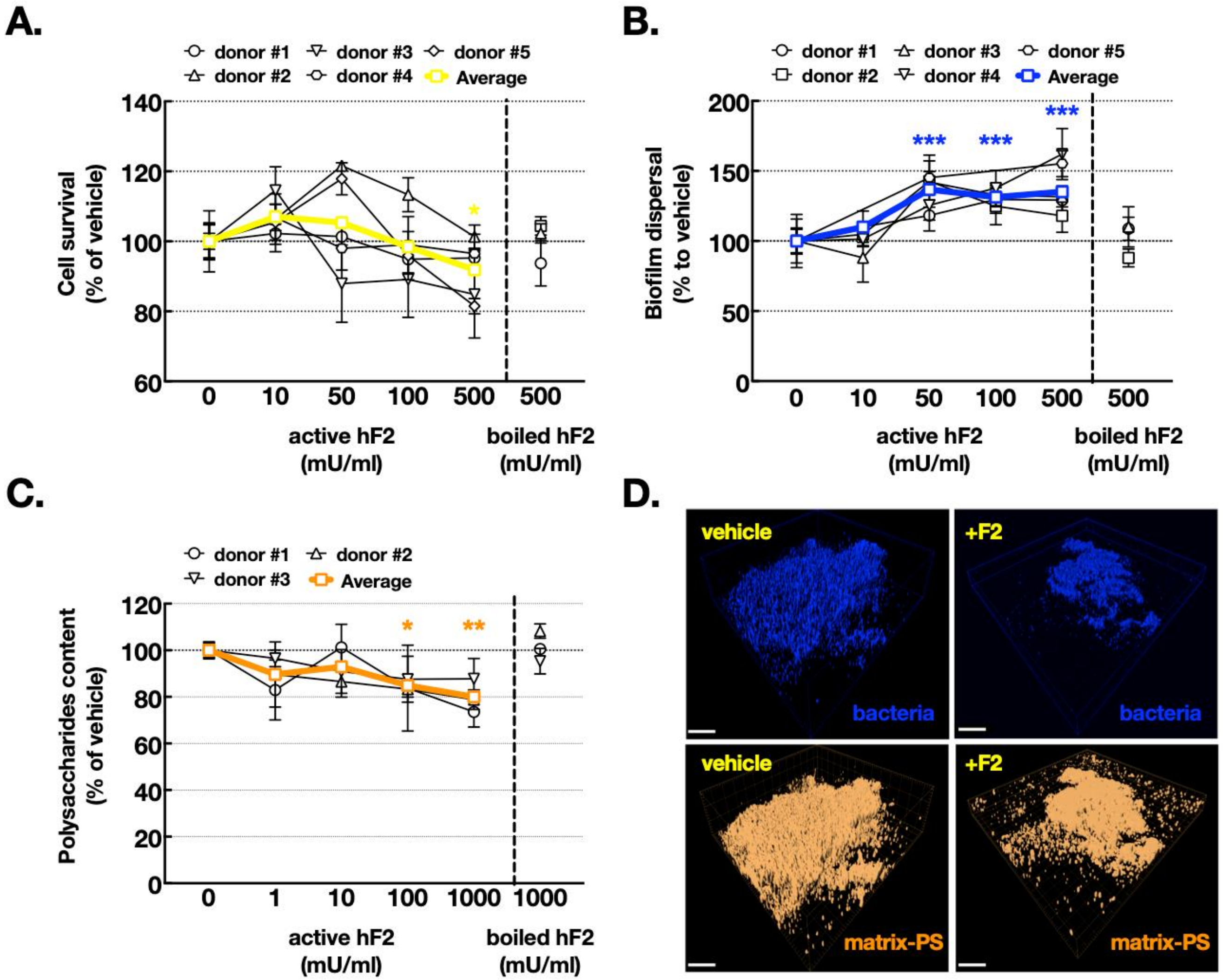

Supplementary Figure 8. High concentrations of thrombin alter matrix-associated polysaccharide content and viability of multispecies anaerobic biofilms. Multispecies anaerobic biofilms were generated from 5 different human healthy donors. Mature biofilms were then exposed to various concentrations of human active thrombin or inactive boiled thrombin for 24 hours. **A.** Biofilm

bacteria survival (rezasurin metabolic assay) was mostly unaffected by thrombin, except at highest concentration (500 $mU.ml^{-1}$, equivalent to 5 nM), and remained unaffected by inactive boiled thrombin (500 $mU.ml^{-1}$, equivalent to 5 nM). **B**. Active, but not boiled, thrombin dose-dependently increases the number of biofilm-dispersed bacteria (optical density at 600 nm). **C.** Concentrations of 100 and 1000 $mU.ml^{-1}$ (equivalent to 1 and 10 nM respectively) of active thrombin reduces the total content of matrix-associated polysaccharides (N-acetyl-glucosamines and sialic acid, wheat germ agglutinin assay). ANOVA analysis with Fisher's LSD test * $P < 0.05$, **$P < 0.01$, ***$P < 0.001$ versus control group. Each concentration corresponds to > 12 biofilms per donor, N=3 independent experiments. **D.** Representative confocal 3D-surfaces reconstructions of human biofilm suggested a limited effect of thrombin (100 $mU.ml^{-1}$, equivalent to 1 nM) on matrix-associated polysaccharides (wheat germ agglutinin stain in orange, PS) and on adherent bacteria (propidium iodide, in blue). Images representative of at least n=3 independent analysis. Scale bars represent 20 µm. Data are presented as mean ± SEM.

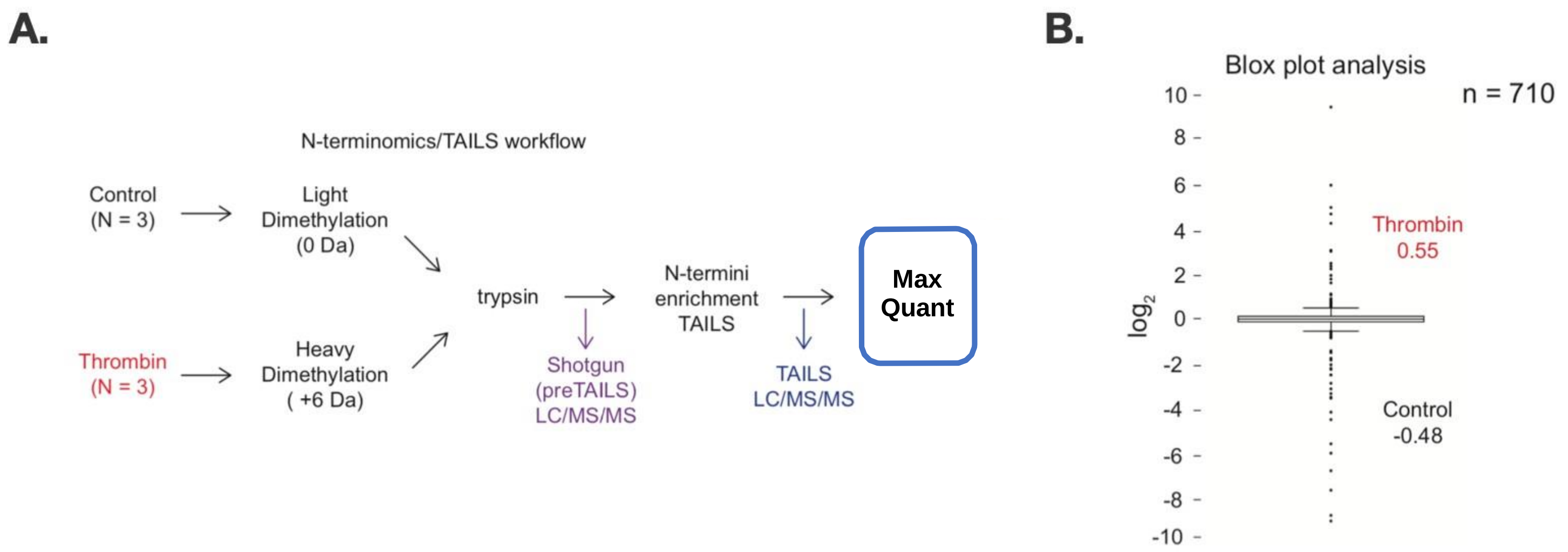

Supplementary Figure 9. N-terminomics/TAILS and shotgun proteomics workflow. A. Schematic representation of N-terminomics/TAILS and shotgun proteomics analysis of three healthy human biofilm samples treated with either vehicle (PBS) or human thrombin (100 mU.ml^{-1} for 24 hours, equivalent to 1 nM). Peptides were identified by LC-MS/MS and analyzed by MaxQuant. **B.** Boxplot analysis of the TAILS peptides (n=710). Significant cut-offs were log2 0.55 (1.46 fold) for the thrombin-treated group and log2 -0.48 (0.51 fold) for the control group.

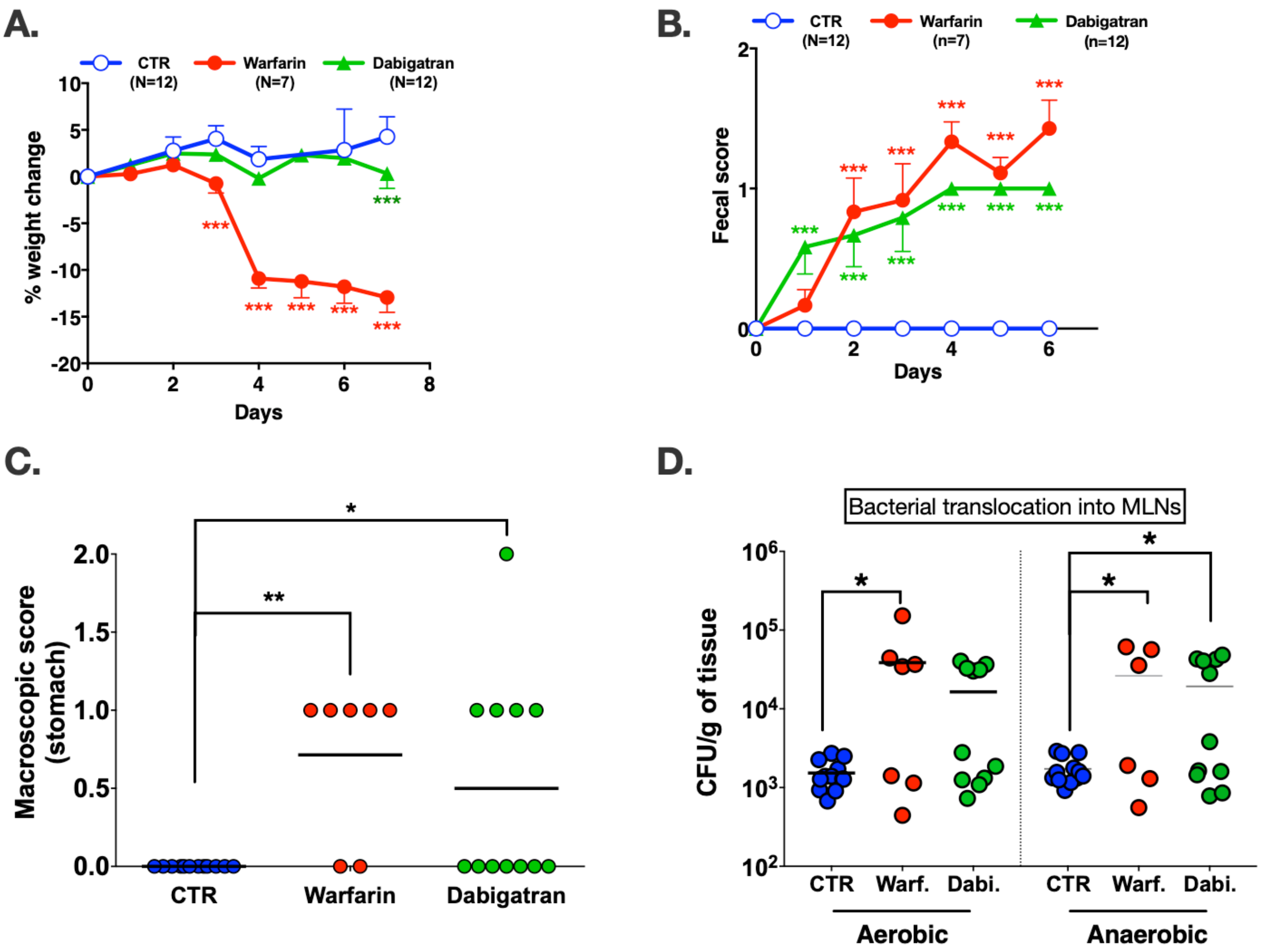

Supplementary Figure 10. Gastrointestinal damage induced by oral anticoagulant treatments Dabigatran (thrombin inhibitor) and warfarin (vitamin K antagonist). C57Bl/6 mice were treated daily with either vehicle (n=12), warfarin (n=7, 10 mg.L^{-1} in drinking water *ad libitum*), or dabigatran etexilate (50 mg/kg/day oral gavage, n=12) for 7 days and euthanized for further analysis (A to D). A. Change

in body weight and **B.** fecal score (consistency and presence of blood) were recorded daily. A &B Data are presented as mean ± SEM ANOVA analysis with Fisher's LSD test * P < 0.05, *** P < 0.001 versus CTR group**. C.** Macroscopic damage score (purpura/petechia, tissue hematoma and edema) in both warfarin and dabigatran etexilate-treated group were significantly greater than the scores measured in vehicle controls. ANOVA test with Fisher's LSD * P < 0.05, ** P < 0.01 versus CTR group. **D.** Bacterial translocation of aerobes (warfarin) and anaerobes (warfarin and dabigatran-etexilate), into mesenteric lymph nodes (MLNs) was significantly greater in animals treated with oral anticoagulants compared to vehicle-treated animals. ANOVA test with Fisher's LSD * P < 0.05 versus CTR group.

A

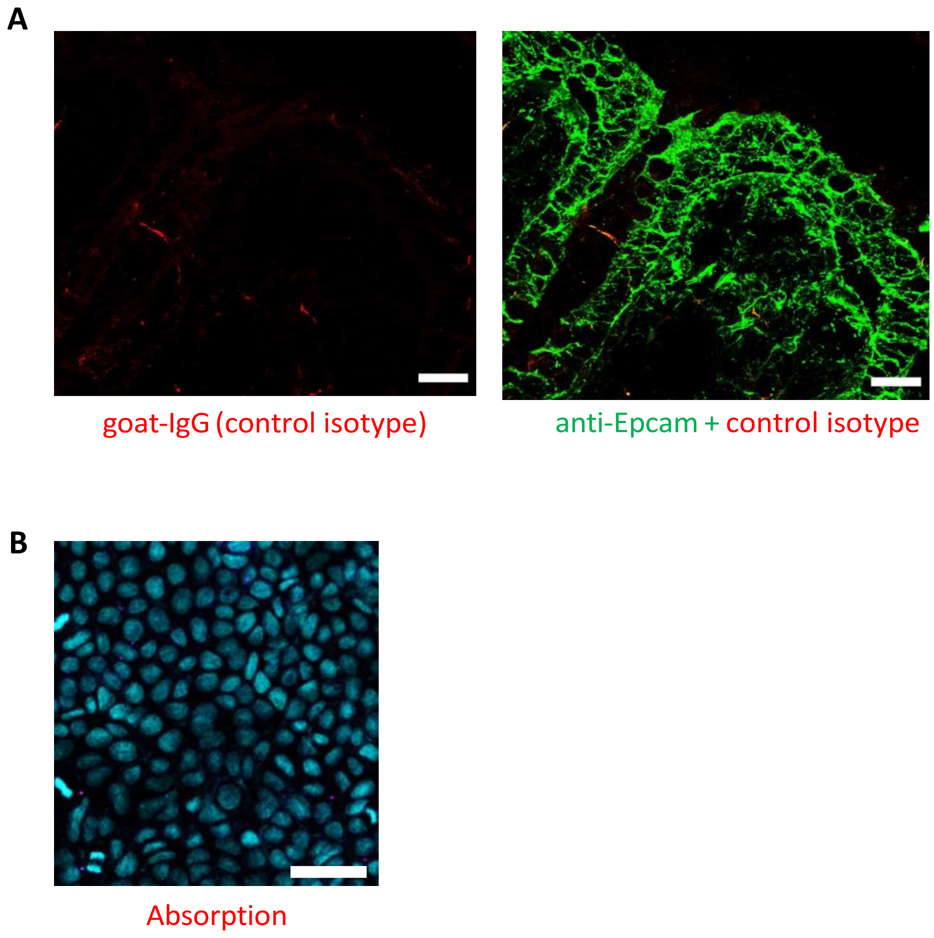

Supplementary Figure 11. Immunostaining controls for thrombin detection by immunofluorescence in human colon tissue and in Caco-2 cells. **A.** Immunofluorescence staining using primary antibody control isotype (goat IgG) demonstrates no unspecific staining in human colonic tissue sections (red). Anti-Epcam antibody was used to stain epithelial cells (green). Scale bar = 20 µm **B.** Immunofluorescence staining of Caco-2 using anti-thrombin IgG pre-absorbed with thrombin antigenic peptide used to raise the anti-thrombin antibody (1:10 Ab:pep ; vol/vol). Nuclei were conterstained with DAPI (blue). Scale bar = 50 µm

Supplementary Table 1. List of PCR primers used in the study.

	Sequence
	**Mouse**
Camp	qMmuCED0004087 PrimePCR Biorad
Tnfa	qMmuCED0004141 PrimePCR Biorad
Il1b	qMmuCID0005641 PrimePCR Biorad
Tff3	qMmuCID0008757 PrimePCR Biorad
Ifng	Forward: CAGCAACAGCAAGGCGAAA<br>Reverse: AGCTCATTGAATGCTTGGCG
Defb4	Forward: TCTGTTTGCATTTCTCCTGGTG<br>Reverse: TTTGCTAAAAGCTGCAGGTGG
Reg3g	Forward: CGACACTGGGCTATGAAC<br>Reverse: TCTCCACTTCAGAAATCCTG
Reg3b	Forward: CTGGTTTGATGCAGAACTG<br>Reverse: TGTTACTCCATTCCCATCC
Muc2	Forward: GTAAACTGCTCTCTGGACTG<br>Reverse: CTTGGAAGACGTGGTAGATG
Zo1	Forward: CGTTATGATCCAGCCCAG Reverse:<br>GCTGGTTTACTCTGAGATGG
Ocln	Forward: ACCCTGACCACTATGAAAC<br>Reverse: CGTCTAGTTCTGCCTGTAAG
Cldn1	Forward: CCTACTTTCCTGCTCCTG Reverse:<br>TGTCCATTTTGTATTTGCTCC
Cldn2	Forward: CCCACAGATACTTGTAAGGAG<br>Reverse: CCAAAAGGCCTAGGATGTAG
Cldn5	Forward: CAATGGCGATTACGACAAG<br>Reverse: GGCTAGTGATGGTCAACG
Cxcl1	Forward: AGCCACACTCAAGAATGGTC<br>Reverse: GTCAGAAGCCAGCGTTCAC
Il17a	Forward: TCCAGAATGTGAAGGTCAACC<br>Reverse: TATCAGGGTCTTCATTGCGG
Nos2	Forward: GCATGTACCCTCAGTTCTG<br>Reverse: ACATCAAAGGTCTCACAGG
Cox2	Forward: TGAGTCATTCACCAGACAG<br>Reverse: GCAGCCATTTCCTTCTCT
F2 (qPCR)	Forward: AACCTGCCCATTGTAGAGCG<br>Reverse: CAGCGGTTGTTAAAGGGGCT
F2 (RT-PCR)	Forward: AACCTGCCCATTGTAGAGCG<br>Reverse: CAGCGGTTGTTAAAGGGGCT
	**Human**
F2 (qPCR)	Forward: TCCTGGGATTGGCTGTGAAC<br>Reverse: CAGCGCATAATGGGTGCTTC
F2 (RT-PCR)	Forward: GAGGACGCCTCGAGATAAGC<br>Reverse: GTGACTTGATCCTGGCCACA
F5 (RT-PCR)	Forward: AATACAGAGGGCAGCAGACA<br>Reverse: CGTCACAGATTCTCCACGCA
F10 (RT-PCR)	Forward: AAACGAGGGTTTCTGTGGTG<br>Reverse: CCCCTACCCTCACCTTGAAT

Supplementary Table 2. Human thrombin cleaves gut biofilm-associated proteins.

Biofilms from three different human healthy donors were treated with vehicle or human thrombin (100 mU.ml^{-1}) for 24 hours. Biofilm-associated proteins were pooled and subjected to an N-terminomics/TAILS and shotgun proteomics analysis. Spectral data were matched to peptide sequences in the UniProt protein database of twelve common intestinal bacterial species using the Andromeda algorithm as implemented in the MaxQuant at a peptide-spectrum match false discovery rate of P< 0.05. Ratio represents peptide enrichment in thrombin-treated *versus* vehicle-treated biofilms.

Peptide sequence	Cleavage site	Uniprot ID [accession code]	Potential protein	Protein function	Potential microbial source	Ratio
SELVESR	^{122}R S^{123}	H1BAZ6;G1VY62;A0A1C7HWV8;A0A1C7HWR8;A0A1C7HTV2;A0A1C7HTR9;A0A1C7HS47;A0A1C7HRQ4;A0A1C7HRH6;A0A1C7HND4;A0A1C7HLT0;H1B7U0 [https://www.uniprot.org/uniprot/H1B7U0]	HMPREF0981_02379	Uncharacterized protein	Erysipelotrichaceae bacterium 6_1_45	64.5
GAEFVARYETDEANVRALDEKMKQFNR	^{225}R G^{226}	A0A1X3C0I5 [https://www.uniprot.org/uniprot/A0A1X3C0I5]	ELS84_0732	ABC superfamily ATP binding cassette transporter, ABC/membrane protein	Enterococcus faecalis (Streptococcus faecalis)	15.3
MLFIEYPTCSTCRKAKEYLQDAG	^{2}R M^{3}	G1VQ68 [https://www.uniprot.org/uniprot/G1VQ68]	HMPREF9022_02149	Uncharacterized protein	Erysipelotrichaceae bacterium 2_2_44A	12.1

MELEIRHIVEETPTVEELR						
MPDGTTGAVSQFEGIPEGGKGAVLDGYYYPNQEGIDFYHR	^{50}R M^{51}	R6UYP4 [https://www.uniprot.org/uniprot/R6UYP4]	BN746_00221	Uncharacterized protein	Erysipelotrichaceae bacterium CAG:64	3.9
KWYVVDATDVPLGR	^{9}R K^{10}	A0A1X3B7M1 [https://www.uniprot.org/uniprot/A0A1X3B7M1]	rplM	50S ribosomal protein L13	Enterococcus faecalis (Streptococcus faecalis)	3.2
KAGHTALTADIDAQKAIYEADVTEGASMHSLAAR	^{29}R K^{30}	A0A2N8HMJ3 [https://www.uniprot.org/uniprot/A0A2N8HMJ3]	CXU19_03050	Uncharacterized protein	Akkermansia muciniphila	2.5
LAESNLR	^{139}R L^{140}	A0A1C7FYY3 [https://www.uniprot.org/uniprot/A0A1C7FYY3]	sigA	RNA polymerase sigma factor SigA	Lachnoclostridium sp. YL32	2.2
NYPGRDAQGAAR	^{66}R N^{67}	A0A2N8HEL8 [https://www.uniprot.org/uniprot/?query=CXU22_04820&sort=score]	CXU22_04820	Uncharacterized protein	Akkermansia muciniphila	2.0
GPVASIYYQGLMR	^{126}R G^{127}	A0A3A9BVA4 [https://www.uniprot.org/uniprot/A0A3A9BVA4]	D7W50_04445	MotA/TolQ/ExbB proton channel family protein	Bacteroides caecimuris	1.9
SKDIEVVYLEDLAAEALINEEVR	^{60}R S^{61}	V7ZMF9 [https://www.uniprot.org/uniprot/V7ZMF9]	arcA	Arginine deiminase	Enterococcus faecalis PF3	1.8

QGISPEMYYQLTGSTEEDLHKQFEGEAETR	^{323}R Q^{324}	V7ZR98 [https://www.uniprot.org/uniprot/V7ZR98]	Tig	Trigger factor	Enterococcus faecalis PF3	1.8
TDIDLPWEQTDKVEALKASLAE	^{372}R T^{373}	V7ZKR0 [https://www.uniprot.org/uniprot/V7ZKR0]	metK	S-adenosylmethionine synthase	Enterococcus faecalis PF3	1.8
DLNNYLNKKLR	^{169}R D^{170}	C7V831 [https://www.uniprot.org/uniprot/C7V831]	EFNG_01499	Aminopeptidase C	Enterococcus faecalis CH188	1.8
SAFTTAAIDLGAHPEYLGANDIQLGKKESVEDTAIVLGSMFDGIEFR	^{61}R S^{62}	R3IEF4 [https://www.uniprot.org/uniprot/R3IEF4]	WOU_00149	Ornithine carbamoyltransferase	Enterococcus faecalis ATCC 6055	1.8
SLLAEKDFTR	^{8}R S^{9}	R3IEF4 [https://www.uniprot.org/uniprot/R3IEF4]	WOU_00149	Ornithine carbamoyltransferase	Enterococcus faecalis ATCC 6055	1.7
ALEEIEAGTVISDPNPEEKR	^{49}R A^{50}	J6R3P9 [https://www.uniprot.org/uniprot/J6R3P9]	rpoZ	DNA-directed RNA polymerase subunit omega	Enterococcus faecalis ERV85	1.7
AAVEEGVVAGGGVALIR	^{404}R A^{405}	A0A1C7HIE3 [https://www.uniprot.org/uniprot/A0A1C7HIE3]	groL	60 kDa chaperonin	Burkholderiales bacterium YL45	1.6
LLFDDIPFLEKAQAEHDAFAELLR	^{36}R L^{37}	V7ZMF9 [https://www.uniprot.org/uniprot/V7ZMF9]	arcA	Arginine deiminase	Enterococcus faecalis PF3	1.5

EFLKERTK GEQTSR	^{254}R E^{255}	A0A1C2G650 [https://www.uniprot.org/uniprot/?query=BFD03_08580&sort=score]	BFD03_08580	Uncharacterized protein	Lactobacillus reuteri	1.5
TDAASIEKI AR	^{235}R T^{236}	V7ZMF9 [https://www.uniprot.org/uniprot/V7ZMF9]	arcA	Arginine deiminase	Enterococcus faecalis PF3	1.5
