# Supplementary material for: Active thrombin produced by the intestinal epithelium controls mucosal biofilms
Source: Nat Commun. 2019 Jul 19;10:3224. doi: 10.1038/s41467-019-11140-w (PMC6642099; doi:10.1038/s41467-019-11140-w)
Supplement: Supplementary file 3 — Reporting Summary [file 41467_2019_11140_MOESM3_ESM.pdf]

nature research

Corresponding author(s): Nathalie Vergnolle

☐ Initial submission ☒ Revised version ☐ Final submission

Reporting Summary

Nature Research wishes to improve the reproducibility of the work that we publish. This form provides structure for consistency and transparency in reporting. For further information on Nature Research policies, see Authors & Referees and the Editorial Policy Checklist.

Please do not complete any field with "not applicable" or n/a. Refer to the help text for what text to use if an item is not relevant to your study.
For final submission: please carefully check your responses for accuracy; you will not be able to make changes later.

Statistical parameters

When statistical analyses are reported, confirm that the following items are present in the relevant location (e.g. figure legend, table legend, main text, or Methods section).

n/a	Confirmed	
☐	☒	The exact sample size (n) for each experimental group/condition, given as a discrete number and unit of measurement
☒	☐	An indication of whether measurements were taken from distinct samples or whether the same sample was measured repeatedly
☐	☒	The statistical test(s) used AND whether they are one- or two-sided<br>*Only common tests should be described solely by name; describe more complex techniques in the Methods section.*
☒	☐	A description of all covariates tested
☐	☒	A description of any assumptions or corrections, such as tests of normality and adjustment for multiple comparisons
☐	☒	A full description of the statistics including central tendency (e.g. means) or other basic estimates (e.g. regression coefficient) AND variation (e.g. standard deviation) or associated estimates of uncertainty (e.g. confidence intervals)
☐	☒	For null hypothesis testing, the test statistic (e.g. F, t, r) with confidence intervals, effect sizes, degrees of freedom and P value noted<br>*Give P values as exact values whenever suitable.*
☒	☐	For Bayesian analysis, information on the choice of priors and Markov chain Monte Carlo settings
☒	☐	For hierarchical and complex designs, identification of the appropriate level for tests and full reporting of outcomes
☒	☐	Estimates of effect sizes (e.g. Cohen's d, Pearson's r), indicating how they were calculated
☐	☒	Clearly defined error bars<br>*State explicitly what error bars represent (e.g. SD, SE, CI)*

Our web collection on statistics for biologists may be useful.

Software and code

Policy information about availability of computer code

Data collection	The 16S bacterial rDNA regions were targeted by the 357wf- 785R primers and analyzed by MiSeq at RTLGenomics (Texas, USA). A complete description of the applied bioinformatic filters is available at www.rtlgenomics.com. RT-PCR bands were blasted on National Center for biotechnology information (blastn NCBI), and aligned using Clustal Omega program.
Data analysis	Hierarchical clustering, PCoA and Permanova analyses were performed using Past 3 freeware (Hammer Ø et al, 2001, Palaeontologia Electronica). Unless otherwise stated, all statistics and graphics were made on GraphPad Prism 6 for Mac.

For manuscripts utilizing custom algorithms or software that are central to the research but not yet described in published literature, software must be made available to editors/reviewers upon request. We strongly encourage code deposition in a community repository (e.g. GitHub). See the Nature Research guidelines for submitting code & software for further information.

Data

Policy information about availability of data

All manuscripts must include a data availability statement. This statement should provide the following information, where applicable:

- Accession codes, unique identifiers, or web links for publicly available datasets
- A list of figures that have associated raw data
- A description of any restrictions on data availability

The raw source data underlying Fig. 1–6 and Supplementary Figs. 1–10 are provided as a Source Data file.
Other data supporting the findings of this manuscript are available from the corresponding authors upon reasonable request.

Field-specific reporting

Please select the best fit for your research. If you are not sure, read the appropriate sections before making your selection.

☒ Life sciences ☐ Behavioural & social sciences ☐ Ecological, evolutionary & environmental sciences

Life sciences study design

All studies must disclose on these points even when the disclosure is negative.

Sample size	We did not performed a statistical analysis to predetermine sample size for mouse study. The number of animal used was based on our extensive experience with this animal model. All sample numbers were stated in figure legends.
Data exclusions	In RT-PCR and western-blots images, visible gaps represent the same gel from which irrelevant lanes were cut out. This is explained in Figure legend. Uncropped blots and gels are presented in the “source data file” with DNA ladder shown. Except for optimization experiments, no other data were excluded.
Replication	All biochemical/microbiological experiments were performed in triplicate, using independent biological samples.
Randomization	Animals and biological samples were randomly allocated.
Blinding	Evaluation of microscopic and macroscopic damage score, as well as the microscopy images acquisition were blindly performed by two skilled experimenter.

Reporting for specific materials, systems and methods

We require information from authors about some types of materials, experimental systems and methods used in many studies. Here, indicate whether each material, system or method listed is relevant to your study. If you are not sure if a list item applies to your research, read the appropriate section before selecting a response.

Materials & experimental systems

n/a	Involved in the study	
☐	☒	Unique biological materials
☐	☒	Antibodies
☐	☒	Eukaryotic cell lines
☒	☐	Palaeontology
☐	☒	Animals and other organisms
☐	☒	Human research participants

Methods

n/a	Involved in the study	
☒	☐	ChIP-seq
☒	☐	Flow cytometry
☒	☐	MRI-based neuroimaging

Unique biological materials

Policy information about availability of materials

Obtaining unique materials	This study involved the use of patient-derived organoids, epithelial cell lines, as well as mucosa-associated microbiota. Ethics Committee approved the human research protocol for the derivation and characterization of these materials (ClinicalTrials.gov Identifier: NCT01990716). All unique materials used are readily available from authors

Antibodies

Antibodies used	All antibodies used in this study are commercially available.
Validation	All antibodies have been previously used on human material and are noted as suitable for this purpose by the distributor.

Eukaryotic cell lines

Policy information about cell lines

Cell line source(s)	Human derived cancer cell lines (Caco-2, HT-29, SW480, HepG2, A549) were purchased from American Type Culture Collection (ATCC).
Authentication	Cell lines used were not authenticated by our laboratory.
Mycoplasma contamination	All cell lines were mycoplasma free, monitored regularly with PCR specific for mycoplasma detection (1 per month)
Commonly misidentified lines (See ICLAC register)	*Name any commonly misidentified cell lines used in the study and provide a rationale for their use.*

Animals and other organisms

Policy information about studies involving animals; ARRIVE guidelines recommended for reporting animal research

Laboratory animals	All mice used in this study were stated in method and figure legend. Conventional and germ- free C57BL/6 mice (10-12 weeks old, males) were used in the present study. Animal procedures were approved by the Laboratory Animal Ethics Committee, in Toulouse and from McMaster University for germ-free animals.
Wild animals	The study did not involve wild animals
Field-collected samples	The study did not involve samples collected from the field

Human research participants

Policy information about studies involving human research participants

Population characteristics	Colon resections from human donors were provided by the Centre Hospitalier de Toulouse (France).
Recruitment	Written and verbal informed consent was obtained before enrollment in the study.

This checklist template is licensed under a Creative Commons Attribution 4.0 International License, which permits use, sharing, adaptation, distribution and reproduction in any medium or format, as long as you give appropriate credit to the original author(s) and the source, provide a link to the Creative Commons license, and indicate if changes were made. The images or other third party material in this article are included in the article's Creative Commons license, unless indicated otherwise in a credit line to the material. If material is not included in the article's Creative Commons license and your intended use is not permitted by statutory regulation or exceeds the permitted use, you will need to obtain permission directly from the copyright holder. To view a copy of this license, visit http://creativecommons.org/licenses/by/4.0/
